# Bioprocess Optimization for the Production of Aromatic Compounds With Metabolically Engineered Hosts: Recent Developments and Future Challenges

**DOI:** 10.3389/fbioe.2020.00096

**Published:** 2020-02-20

**Authors:** Adelaide Braga, Nuno Faria

**Affiliations:** Centre of Biological Engineering, University of Minho, Braga, Portugal

**Keywords:** aromatic compounds, metabolic engineering, microorganisms, process optimization, synthetic biology, shikimate pathway

## Abstract

The most common route to produce aromatic chemicals – organic compounds containing at least one benzene ring in their structure – is chemical synthesis. These processes, usually starting from an extracted fossil oil molecule such as benzene, toluene, or xylene, are highly environmentally unfriendly due to the use of non-renewable raw materials, high energy consumption and the usual production of toxic by-products. An alternative way to produce aromatic compounds is extraction from plants. These extractions typically have a low yield and a high purification cost. This motivates the search for alternative platforms to produce aromatic compounds through low-cost and environmentally friendly processes. Microorganisms are able to synthesize aromatic amino acids through the shikimate pathway. The construction of microbial cell factories able to produce the desired molecule from renewable feedstock becomes a promising alternative. This review article focuses on the recent advances in microbial production of aromatic products, with a special emphasis on metabolic engineering strategies, as well as bioprocess optimization. The recent combination of these two techniques has resulted in the development of several alternative processes to produce phenylpropanoids, aromatic alcohols, phenolic aldehydes, and others. Chemical species that were unavailable for human consumption due to the high cost and/or high environmental impact of their production, have now become accessible.

## Introduction

The increasing demand for “natural” labeled products, the adoption of a healthy life style associated with growing concerns about global warming and limited supplies of fossil fuels, promote the development of alternative ways for producing fuels and commodity chemicals using renewable feedstocks in eco-friendly processes. In this scenario, the use of biotechnological platforms for their production is becoming a promising alternative (Sun et al., 2015; Braga et al., 2018a; Milke et al., 2018; Park et al., 2018).

An important class of petrochemical compounds that have been considered as promising targets for biotechnological production are aromatic compounds (Knaggs, 2003; Lee and Wendisch, 2017; Noda and Kondo, 2017). They are typically produced employing fossil feedstocks as raw materials and have a wide range of industrial and commercial applications as building blocks for the synthesis of polymer materials like functional plastics and fibers, food and feed additives, nutraceuticals and pharmaceuticals (Krömer et al., 2013; Averesch and Krömer, 2018). The economic importance of these compounds is quite significant; in 2017 their global market size was USD185.9 billion and it is expected that, in 2025, their global production volume will reach 168,733.35 thousand tons (Averesch and Kayser, 2014), with the demand for aromatic compounds for gasoline, pharmaceuticals and detergents as main driving force.

In the last decades, microorganisms have emerged as attractive platforms for producing former petroleum-derived compounds from renewable starting materials (Borodina and Nielsen, 2014; Krivoruchko and Nielsen, 2015; Noda and Kondo, 2017). Until now, several derivatives of BTX (benzene, toluene, and the three isomers of xylene), such as styrene, hydroxystyrene, phenol and vanillin, have been produced using microbial hosts by direct bioconversion of precursors or via *de novo* synthesis (Wierckx et al., 2005; Vannelli et al., 2007; Mckenna and Nielsen, 2011; Ni et al., 2015). However, only a few compounds, such as vanillin and resveratrol, have reached bio-based production at commercial scale (Nakamura and Whited, 2003; Yim et al., 2011; Paddon et al., 2013; Van Dien, 2013). Nevertheless, despite the efforts that have been made until now, the production of benzene, toluene or xylene in a renewable way has not been reported.

Microorganisms can grow with high growth rates and achieve high biomass yields, in scalable cultivation and production processes. They are also able to grow in diverse media, from abundant and inexpensive feedstocks. However, they do not naturally (over-)produce these compounds or, if they do, the yields are very low. In order to enable production, it is necessary to functionally integrate heterologous pathways or genetically modify the microbial hosts (Rodrigues et al., 2015; Chouhan et al., 2017; Gottardi et al., 2017; Milke et al., 2018; Wang J. et al., 2018). Aromatic compounds are produced by microbial hosts via the shikimate pathway, which leads to the production of aromatic amino acids as well as other aromatic precursors (Herrmann, 1995; Maeda and Dudareva, 2012; Averesch and Krömer, 2018). This can be achieved by the functional reconstruction of naturally occurring pathways or by *de novo* pathway engineering (Dhamankar and Prather, 2011; Wu et al., 2018). *Escherichia coli* and *Saccharomyces cerevisiae* are the most commonly employed microorganisms for aromatic compound production. However, more recently, other hosts have also been explored due to their peculiarities, such as *Corynebacterium glutamicum, Lactococcus lactis, Pseudomonas putida*, and *Streptomyces lividans* (Sachan et al., 2006; Gosset, 2009; Verhoef et al., 2009; Gaspar et al., 2016; Kallscheuer et al., 2016, 2019; Dudnik et al., 2017, 2018; Braga et al., 2018a; Tilburg et al., 2019).

This review presents an overview of recent advances in microbial production of the most relevant aromatic compounds, including vanillin, salicylic acid, *p*-hydroxybenzoic acid and others strategies for strain design are compared with an emphasis on the development of biosynthetic pathways, the application of protein engineering, carbon flux redirection, use of alternative substrates, engineering substrate uptake and optimization of culture conditions. We present and explain some of the current challenges and gaps that in our knowledge, must be overcome in order to render the biotechnological production of aromatic compounds, in an attractive and feasible way for the commercial scale. Table 1 presents a summary of the recent reports (last 4 years) regarding the production of aromatic compounds in engineered microbial hosts, comparing the used carbon source, organism and strain, (over-)expressed and/or knocked out genes.

**TABLE 1:** Summary of the literature on the production titer of some aromatic compounds obtained through metabolic engineering in microorganisms, in the last 4 years.

**Compound**	**Microorganism**	**Heterologous enzymes (source)**	**Substrate/precursor**	**Titer (mg L**^–^**^1^)**	**References**
Salicylic acid	*Escherichia coli*	*pykF*, *pykA pheA*, *tyrA* deleted *menF* (*E. coli) pchB* (*Pseudomonas aeruginosa*)	Glucose	11500	Noda et al., 2016
*p*-Hydroxybenzoate	*Corynebacterium glutamicum*	*xylA*, *xylB*, bglF, bglA, aroA, aroD, *aroE*, *aroCKB*, *araBAD*, *araE*, *tkt*, *tal* overexpressed *aroG* (*E. coli*) *ubi*C (*Providencia rustigianii*) *ldhA*, *qsuB*, *qsuD*, *pobA*, *poxF*, *pyk*, *hd*pA deleted	Glucose	36600	Kitade et al., 2018
	*Pseudomonas putida*	*UbiC* (*E. coli*) *ar*oG^fbr^ *pobA*, *phA*, *trpE*, *hexR* deleted	Glucose	1730	Krömer, 2016
	*S. cerevisiae*	Overexpression *ARO4*^K229L^ *aroL*, *ubiC* (*E. coli*) *ARO4*^fbr^*ARO7*, *TRP3* deleted	Glucose	2900	Averesch et al., 2017
	*P. taiwanensis* VLB120	*pobA* and *hpd* deleted *fcs*, *ech* and *vhd* (*P. putida* S12) PAL (*Rhodosporidium toruloides*) *ar*oG^fbr^ and *try*^fbr^ overexpression *ppsA* and *pgi*	Glycerol	9900	Lenzen et al., 2019
*p*-Coumaric acid	*E. coli*	C4H (*Lycoris aurea*) PAL (*Arabidopsis thaliana*) overexpression *pntAB*	Glucose	25.6	Li et al., 2018
	*S. cerevisiae*	*Aro10*, *pdc5* deleted TAL (*Flavobacterium johnsoniaeu*) *Aro7^fbr^ and Aro4^fbr^ aroL* (*E. coli*)	Xylose	242	Borja et al., 2019
	*E. coli BL21 (DE3)*	TAL (*Saccharothrix espanaensis) tyrR* and *pheA* deleted *aroG*^fbr^ and *tyrA*^fbr^	Glucose	100.1	Gyuun et al., 2016
	*S. cerevisiae*	*Aro10*, *pdc5* deleted TAL (*Flavobacterium johnsoniaeu*) *Aro7^G141^ and Aro4^K229^ aroL* (*E. coli*)	Glucose	2400	Rodriguez et al., 2017
Caffeic acid	*E. coli BL21 (DE3)*	*tyrR* and *tyrA* deleted TAL (*Saccharothrix espanaensis) aroG*^fbr^ and *tyrA*^fbr^ *sam5* (*S. espanaensis)*	Glucose	138.2	Gyuun et al., 2016
	*S. cerevisiae*	Codon optimized *hpaB* (*P. aeruginosa*) Codon optimized *hpaC* (*Salmonella entérica*) TAL (*Rhodosporidium toruloides*)	L-Tyr	289.4	Liu et al., 2019a
	*E. coli*	*tryR* deleted *tyrA*^fbr^ *aroG*^fbr^ *tktA* and *ppsA* overexpressed *hpaBC* (*P. aeruginosa*) f*evV* (*Streptomyces* sp. WK-5344)	Kraft pulp	233	Kawaguchi et al., 2017
2-Phenylethanol	*E. coli*	*ar*oG^fbr^ and *pheA*^fbr^ *kdc* (*S. cerevisiae)* overexpression *yigB Aro8* (*S. cerevisiae)*	Glucose	1016	Liu et al., 2018
	*S. cerevisiae* YS58	*ARO8* and *ARO10* overexpressed	L-Phe	3200	Wang et al., 2017
	*E. coli*	*aroG*^fbr^ and *pheA*^fbr^ *kdc* (*S. cerevisiae* YPH499) *yigB* overexpressed *aro8* (*S. cerevisiae)*	Glucose	1000	Guo et al., 2018
	*S. cerevisiae*	*Gap1*, *ARO8*, *ARO10*, *Adh2*, *Gdh2* overexpressed	Glucose	6300	Wang Z. et al., 2018
Vanillin	*E. coli top 10*	*fcs* and *ech* (*Amycolatopsis* sp. HR)	Ferulic acid	68	Chakraborty et al., 2016
	*E. coli*	*fcs* and *ech* (*P. fluorescens* BF13)	Ferulic acid	4258.8	Luziatelli et al., 2019

*frb, feedback-resistant; TAL, tyrosine ammonia lyase; PAL, phenylalanine ammonia lyase.*

## The Shikimate Pathway: a Path for Aromatic Compounds Production

**FIGURE 1:**
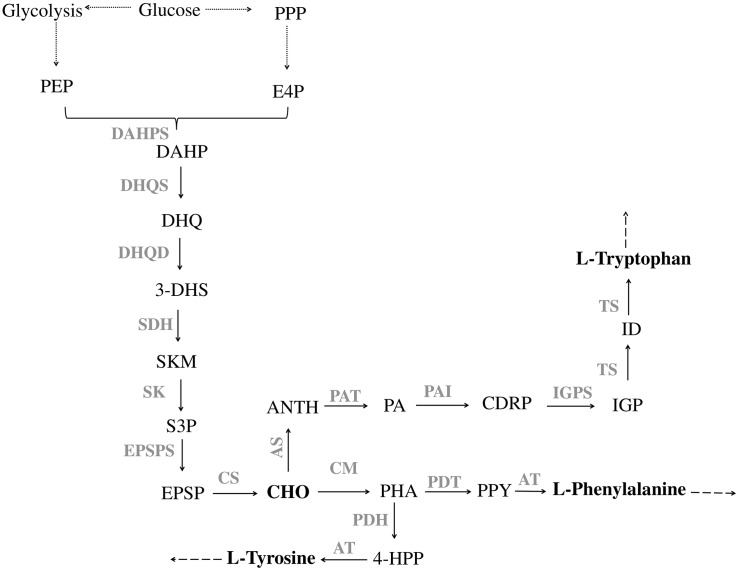
Pathway of aromatic amino acid biosynthesis. PPP, pentose phosphate pathway; E4P, erythrose 4-phosphate; PEP, phosphoenolpyruvate; DAHPS, DAHP synthase; DAHP, 3-Deoxy-D-arabinoheptulosonate 7-phosphate; DHQS, 3-dehydroquinate synthase; DHQ, 3-dehydroquinate; DHQD, 3-dehydroquinate dehydratase; 3-DHS, 3-dehydroshikimate; SDH, shikimate 5-dehydrogenase; SKM, shikimate; SK, shikimate kinase; S3P, shikimate 3-phosphate; EPSPS, 5-enolpyruvylshikimate 3-phosphate synthase; EPSP, 5-enolpyruvylshikimate-3-phosphate; CS, chorismate synthase; CHO, chorismate; CM, chorismate mutase; PHA, prephenate; PDH, prephenate dehydrogenase; 4-HPP, 4-hydroxyphenylpyruvate; AT, aminotransferase; PDT, prephenate dehydratase; PPY, phenylpyruvate; AS, anthranilate synthase; ANTH, anthranilate; PAT, phosphoribosylanthranilate transferase; PA, phosphoribosylanthranilate; PAI, phosphoribosylanthranilate isomerase; CDRP, l-(*O*-carboxyphenylamino)-l-deoxyribulose-5-phosphate; IGPS, indole-3-glycerol phosphate synthase; IGP, indole-3-glycerol phosphate; TS, tryptophan synthase; ID, indole. Solid lines indicate a single step; dotted lines indicate multiple steps.

In microorganisms, the production of aromatic compounds is almost always obtained via the shikimate (SKM) pathway. This route leads to the biosynthesis of aromatic amino acids, L-tyrosine (L-Tyr), L-tryptophan (L-Trp) and L-phenylalanine (L-Phe), and a wide range of aromatic precursors (Knaggs, 2003; Noda et al., 2016; Lai et al., 2017). The first reaction in the shikimate pathway is the condensation of the central carbon metabolism intermediates, phosphoenolpyruvate (PEP) and erythrose-4-phosphate (E4P), to yield 3-deoxy-D-arabino-heptulosonate-7-phosphate (DAHP). After that, six successive enzymatic reactions lead to the production of chorismate (CHO), the end product of the SKM pathway (Figure 1) and the starter unit for the production of aromatic amino acids as well as different aromatic compounds (phenylpropanoids, salicylic acid, *p*-hydroxybenzoic acid, aromatic alcohols, vanillin, among others) (Noda et al., 2016).

The first step for L-Phe and L-Tyr production is catalyzed by chorismate mutase (CM), which converts CHO to prephenate (PHA). After that, PHA undergoes decarboxylation and dehydration yielding phenylpyruvate (PPY) or is oxidatively decarboxylated to 4-hydroxyphenylpyruvate (4-HPP). The reactions are catalyzed by prephenate dehydratase (PDT) and prephenate dehydrogenase (PDH), respectively. The last step comprises the transamination of PPY to L-Phe and of 4-HPP to L-Tyr that is catalyzed by an aminotransferase (AT) (Tzin et al., 2001; Figure 1). The pathway for L-Trp production from CHO requires six steps. The first one is catalyzed by anthranilate synthase (AS) that converts CHO to anthranilate (ANTH), which is further converted to phosphoribosylanthranilate (PA) by anthranilate phosphoribosyl transferase (PAT). The third step in this pathway leads to the production of l-(*O*-carboxyphenylamino)-l-deoxyribulose-5–phosphate (CDRP) by phosphoribosylanthranilate isomerase (PAI). The fourth enzyme of L-Trp biosynthesis is indole-3-glycerol phosphate synthase (IGPS), which catalyzes the conversion of CDRP to indole-3-glycerol phosphate (IGP). In the last two steps, IGP is cleaved by tryptophan synthase (TS) into indole (ID) that is ligated to L-serine to yield L-Trp (Figure 1; Priya et al., 2014).

One of the main bottlenecks in the microbial production of aromatic compounds is the availability of the precursors PEP (produced during glycolysis) and E4P (derived from the pentose phosphate pathway – PPP) (Suástegui et al., 2016; Noda and Kondo, 2017; Averesch and Krömer, 2018; Wu et al., 2018). Different strategies have been described in order to engineer the central carbon metabolism into this direction (Leonard et al., 2005; Papagianni, 2012; Nielsen and Keasling, 2016). In fact, the available fluxes of both precursors differ considerably. Suástegui et al. (2016) studied the E4P and PEP flux in *S. cerevisiae* using metabolic flux analysis and observed that E4P was clearly the limiting precursor. Therefore, establishing a balance between the ratio of both precursors and increasing their availability appeared to be the two main strategies to follow in order to increase aromatic compounds production.

E4P can be produced from PPP or from sedoheptulose-1,7-bisphosphate in a reaction that is probably favored when the intracellular levels of sedoheptulose-7-phosphate (S7P)are high (Nagy and Haschemi, 2013). S7P is an intermediate in non-oxidative part of PPP, that is produced from xylulose 5-phosphate and ribose 5-phosphate by transketolase. The most common approaches to increase E4P production are the overexpression of transaldolase and transketolase genes, to promote the conversion of S7P and glyceraldehyde-3-phosphate (G3P) to E4P and fructose 6-phosphate (F6P) (Bongaerts et al., 2001; Noda and Kondo, 2017; Averesch and Krömer, 2018) and to enhance the supply of E4P (Lütke-Eversloh and Stephanopoulos, 2007; Bulter et al., 2003). Following this strategy, Knop et al. (2001) observed an increase in the shikimic acid titer, an intermediate of the SMK pathway, from 38 to 52 g L^–1^, after overexpression of transketolase gene (*tktA*) in *E. coli*. The role of transaldolase for the production of the PPP was analyzed by Lu and Liao (1997) and Sprenger et al. (1998). They observed that the overexpression of *talB* increases the production of DAHP from glucose. Moreover, Lu and Liao (1997) concluded that transketolase is more effective in directing the carbon flux to the aromatic pathway than transaldolase. In fact, the overexpression of the transaldolase gene in strains which already overexpress the transketolase gene did not show a further increase in production of aromatic compounds. This result may be related with the saturation of E4P supply when *tktA* was overexpressed (Lu and Liao, 1997). Nevertheless, the overexpression of the transketolase gene proved to have a limited impact in the E4P poll which can be correlated with the preference of this enzyme for catalyzing the E4P consuming reaction (Curran et al., 2013). Other efforts to increase the carbon flux in the PPP include the overexpression of the gene coding for glucose-6-phosphate dehydrogenase, that has been shown to increase the availability of ribulose-5-phosphate (R5P) and E4P (Yakandawala et al., 2008; Rodriguez et al., 2013) or the deletion of genes that encode the phosphoglucose isomerase, that forces the cell to metabolize the substrate completely via PPP (Mascarenhas et al., 1991). However, the later approach blocks the oxidative shunt of the PPP, which is the main source of the redox cofactor NADPH, required by the shikimate dehydrogenase as well as by many enzymes in downstream pathways (Zhang J. et al., 2015). The use of other carbon sources that have different transporters, such as hexoses (as sucrose and gluconate), pentoses (xylose and arabinose) and glycerol (Kai Li and Frost, 1999; Ahn et al., 2008; Martínez et al., 2008; Chen et al., 2012), is also an alternative way to increase the E4P poll. In the last year, Liu et al. (2019b) proposed a different strategy to increase the scarcity of E4P in *S. cerevisiae*. They investigated a heterologous phosphoketolase (PHK) pathway, including a phosphoketolase from *Bifidobacterium breve* (*Bbxfpk*) and a phosphotransacetylase from *Clostridium kluyveri* (*Ckpta*). Phosphoketolase is able to split fructose-6-phosphate into E4P and acetyl-phosphate, and the introduction of this pathway could, theoretically, divert part of the carbon flux from glycolysis directly toward E4P. The authors observed a 5.4-fold enhance in E4P concentration in the *BbXfpk*-expressing strain. When compared with the overexpression of the transketolase-encoding gene, this approach resulted in a lower E4P availability (Liu et al., 2019b). However, none of these strategies were able to efficiently divert carbon flux from glycolysis toward E4P, to provide sufficient levels for the biosynthesis of aromatic compounds.

The availability of the other precursor, PEP, is also an important factor that needs to be considered when designing a strategy to construct a strain able to produce aromatic compounds (Rodriguez et al., 2013; Noda and Kondo, 2017). PEP is required for the simultaneous uptake and phosphorylation of glucose (PEP:glucose phosphotransferase system – PTS) and it is also involved in reactions catalyzed by the enzymes phosphoenolpyruvate carboxylase and pyruvate kinase that catalyzes the ATP-producing conversion of PEP to pyruvate. The glucose transport by PTS is the main PEP consuming activity and for this reason the construction of PTS-deficient strains is one of the most common approaches to increase PEP availability and therefore, aromatic compounds yield from glucose (Gu et al., 2012, 2013). However, the main problem of this strategy is the low cellular growth rate. The use of a non-PTS system which does not consume PEP is an alternative way to allow high PEP availability (Sprenger et al., 1998; Chandran et al., 2003). For example, glucose can be transported by galactose permease (encoded by *galP*) and further phosphorylated by glucokinase (encoded by *glk*) (Yi et al., 2003; Balderas-hernández et al., 2009). Additionally, Yi et al. (2003) described the utilization of a glucose facilitator from *Zymomonas mobilis* (encoded by *glf*), that transports glucose by facilitated diffusion, in combination with plasmid-localized *Z. mobilis glk* (encoded glucokinase), attaining a 3-dehydroshikimic acid (a key intermediate for aromatic compounds production) production of 60 g L^–1^, in *E. coli*. In 2011, Ikeda et al. (2011) identified a new non-PTS system, a *myo*-inositol-induced transporter (encoded by *iolT1*) in *C. glutamicum*. Furthermore, an increase in the PEP availability has been achieved by modulation of the carbon flux from PEP to the tricarboxylic acid cycle (TCA) by inactivation of pyruvate kinase genes (Gosset et al., 1996; Chandran et al., 2003; Escalante et al., 2010) and PEP carboxylase (Tan et al., 2013). On the other hand, the overexpression of the genes that encode PEP synthetase which catalyzes the conversion of pyruvate into PEP, enhanced its level (Patnaik and Liao, 1994; Tatarko and Romeo, 2001). PEP carboxykinase catalyzes the formation of PEP from oxaloacetate. The overexpression of the gene that encodes it – *pckA* – has also been proposed as a strategy to increase the yield of aromatic amino acids (Gulevich et al., 2006). An interesting approach to enhance the PEP availability is the attenuation of CsrA, a protein that regulates transcription of genes involved in carbon metabolism and energy metabolism (Wang et al., 2013). It was found that the absence of CsrA could enhance the metabolic flow of gluconeogenesis, contributing to the accumulation of PEP. Tatarko and Romeo (2001) knocked out the *csraA* gene and observed an increase in the PEP concentration. The overexpression of *csrB*, a small untranslated RNA from the carbon storage regulator, also improves the availability of PEP in *E. coli* (Yakandawala et al., 2008).

Notwithstanding the progress achieved, the industrial application of these strategies still poses some problems. The redirection of the carbon flux into a desired pathway usually results in a reduced cell growth rate and/or production of unwanted by-products (Patnaik et al., 1992) which usually ruins the economic viability of an eventual industrial process as will be further discussed (section “Discussion”).

After the establishment of an adequate supply of precursors it is essential to redirect this carbon toward the SKM pathway and remove limiting steps to increase the production of target compounds. The SKM pathway is highly complex. It is mainly regulated at the transcription and enzymatic activity level. As previously described, the first step of the SKM pathway is the DAHP production, catalyzed by DAHP synthases. This is one of the most strictly regulated steps in this route (Figure 1). In fact, DAHP synthase activity is regulated by the concentration of the downstream reaction products of the SKM pathway, the aromatic amino acids. This mechanism is a clever way for cells to make just the right amount of product. When the concentration of aromatic amino acids is high, they will block the DAHP synthase activity, preventing its production until the existing supply has been used up (Bongaerts et al., 2001; Gosset, 2009; Gottardi et al., 2017). In *S. cerevisiae*, two DAHP synthase isozymes (encoded by *ARO3* and *ARO4* genes) are feedback inhibited by L-Phe and L-Tyr, respectively (Paravicini et al., 1989). *E. coli* has three different DAHP synthase isozymes (encoded by *aroF, aroG, aroH*), and each one is vulnerable to inhibition by an aromatic amino acids: L-Phe, L-Tyr and L-Trp, respectively (Hu et al., 2003; Gu et al., 2012). In addition to the allosteric inhibition, it is also necessary to take into account the transcriptional repression mediated by the protein TyrR (tyrosine repressor). This can repress *aroF* and *aroG*, whereas the transcription of *aroH* is controlled by the protein TrpP (tryptophan repressor) (Pittard et al., 2005; Keseler et al., 2013). In *C. glutamicum*, the DAHP synthase isozymes are encoded by *aroG* and *aroF. AroG* is feedback inhibited by L-Phe, chorismate and prephenate, whereas *aroF* is feedback inhibited by L-Tyr and L-Trp (Lee et al., 2009).

To overcome this natural limitation, different strategies have been described, such as the use of DAHP synthase which is not sensitive to feedback-inhibition (feedback-resistant – fbr) (Frost and Draths, 1995). Shumilin et al. (1999) determined a 3D structure of DAHP synthase co-crystallized with PEP, demonstrating the possible nine binding sites of L-Phe for feedback-inhibition. Random or directed mutagenesis at these specific amino acids residues, such as Asp146Asn, and Pro150Leu (Kikuchi et al., 1997), is the most common approach used to generate feedback-resistant variants of DAHP synthase. Hartmann et al. (2003) determined the crystal structure of Aro4p and demonstrated that with a single lysine-to-leucine substitution at position 229, the protein is L-Phe and L-Tyr insensitive. In combination with the deletion of *ARO3*, this strategy led to a 4-fold increase in the flux through the aromatic amino acid-forming pathway (Luttik et al., 2008). Similarly, the introduction of a tyrosine-insensitive *ARO4* allele (*ARO4* ^G226S^) was also reported by Schnappauf et al. (1998). In *E. coli* a similar approach was also described with the introduction of feedback-resistant derivatives of *aroF*^fbr^ and *aroG*^fbr^, using either plasmids or chromosomal integration for expression of the modified encoding genes (Ger et al., 1994; Jossek et al., 2001). In this context, the reactions catalyzed by 3-dehydroquinate synthase, shikimate kinase and shikimate 5-dehydrogenase are also considered rate-limiting (Dell and Frost, 1993; Kramer et al., 2003; Oldiges et al., 2004; Juminaga et al., 2012). Different strategies have been applied to overcome these limitations, such as: the overexpression of the genes that encode these enzymes by plasmid-cloned genes, their chromosomal integration, promoter engineering by chromosomal evolution, or co-expression of the genes in a modular operon under control of diverse promoters (Chandran et al., 2003; Lütke-Eversloh and Stephanopoulos, 2005; Escalante et al., 2010; Rodriguez et al., 2013; Cui et al., 2014).

Another regulatory point is present at the chorismate branch, at which the chorismate mutase and prephenate dehydratase are feedback regulated by the end products, L-Phe and L-Try (Lütke-Eversloh and Stephanopoulos, 2005; Reifenrath et al., 2018). The most common strategies to overcome this bottleneck are the application of mutations that confer feedback resistance to chorismate mutase-prephenate dehydratase or the utilization of evolved genes (*pheA*^ev^) (Báez-Viveros et al., 2004; Lütke-Eversloh and Stephanopoulos, 2005; Ikeda, 2006; Sprenger, 2007; Luttik et al., 2008). Backman et al. (1990) used a recombinant *E. coli* strain carrying *pheA*^fbr^ and *aroF*^fbr^ for L-Phe production and achieved a titer of 50 g L^–1^ with a yield of 0.25 (mol L-Phe mol glucose^–1^) after 36 h. This is the highest titer reported so far. Furthermore, Báez-Viveros et al. (2004) observed that the overexpression of evolved genes (*pheA*^ev^) had a positive and significant impact on L-Phe production in *E. coli*, showing a 3–4-fold improvement, when compared with equivalent strains expressing *pheA*^fbr^. The use of L-Tyr- or L-Phe-overproducing strains for the production of some aromatic compounds, derived from aromatic amino acids, has been reported by many authors. For their construction, the most common approaches include overexpression of *aroG*^fbr^ and *tyrA*^fbr^ and in some cases *ppsA, tktA* and the deletion of *tyrR* (Kang et al., 2012; Lin and Yan, 2012; Santos et al., 2012; Huang et al., 2013), achieving L-Phe and L-Try titers of 50 and 55 g L^–1^, respectively (Patnaik and Liao, 1994; Ikeda, 2006; Sprenger, 2007). However, most of the studies that have been performed, reported the expression and/or regulation of key genes, under the control of constitutively expressed or inducible promoters in plasmid-cloned operons. Nevertheless, this approach has several drawbacks, ranging from structural and segregational instability to metabolic burden of plasmid replication (Noack et al., 1981; Bentley et al., 1990). To overcome these drawbacks, Cui et al. (2014) developed a plasmid free methodology for shikimic acid production, an important intermediate of the SKM pathway, in *E. coli*. *AroG*^fbr^, *aroB, aroE*, and *tktA* genes were chromosomally integrated by tuning the copy number and expression using chemically induced chromosomal evolution with triclosan. They also overexpressed the *ppsA* and *csrB* genes to enhance the PEP/pyruvate pool. Finally, *pntAB* or *nadK* genes were also chromosomally overexpressed in order to increase the NADPH pull. The final strain was able to produce 3.12 g L^–1^ of shikimic acid with a glucose yield of 0.33 mol mol^–1^. They also demonstrated that the overexpression of *pntAB* or *nadK* genes increase the NADPH availability. This is the first report of an engineered shikimic acid producing strain of *E. coli* that lacks both a plasmid and an antibiotic marker.

Despite the efforts that have been made, it is also important to study different strategies to minimize carbon loss to competing pathways. Gu et al. (2012) reported an increase in L-Trp concentration after a knock out in the gene *tnaA*, which codes for a tryptophanase to avoid product degradation. On the other hand, the modification of aromatic compounds transport system, as the inactivation of permease genes *aroP*, *mtr* and *tnaB* to avoid product re-internalization, or the overexpression of genes that encodes exporter proteins (e.g., *yddG*), can also be used as interesting approaches to increase its production (Liu et al., 2012; Wang et al., 2013). Rodriguez et al. (2013) demonstrated that the inactivation of *ydiB* (coding for shikimate dehydrogenase/quinate dehydrogenase) leads to a decrease in byproduct formation, improving the carbon flux toward the desired aromatic compound production.

## Strategies for Production of Aromatic Compounds

In the last decade, several attempts to implement the production of aromatic compounds in cells have been reported (Bongaerts et al., 2001; Dias et al., 2017; Lee and Wendisch, 2017; Beata et al., 2019). The first studies focused on the identification of the microorganisms that are able to produce, natively, aromatic compounds, such as 2-phenylethanol, and/or metabolites that are biosynthetic precursors or derivatives of aromatic compounds, such as SKM, chorismate (CHO), and aromatic amino acids (L-Phe, L-Tyr and Trp), with high efficiency. Then, engineered strains were developed and the production processes optimized in order to raise the product titer to g L^–1^-scale. Nowadays, microbial hosts are able to produce a large spectrum of target products, including chemicals they do not naturally produce (Pandey et al., 2016; Wang et al., 2016).

In this section, we will focus on illustrating the current strategies described for producing aromatic compounds, beginning with the products that are considered industrial building blocks, as salicylic acid, *p*-hydroxybenzoic acid, *p*-coumaric acid, cinnamic acid, ferulic acid and 2-phenylethanol. A brief overview of some relevant aromatic compounds that are widely used as fine chemicals, such as vanillin, will be further presented.

**FIGURE 2:**
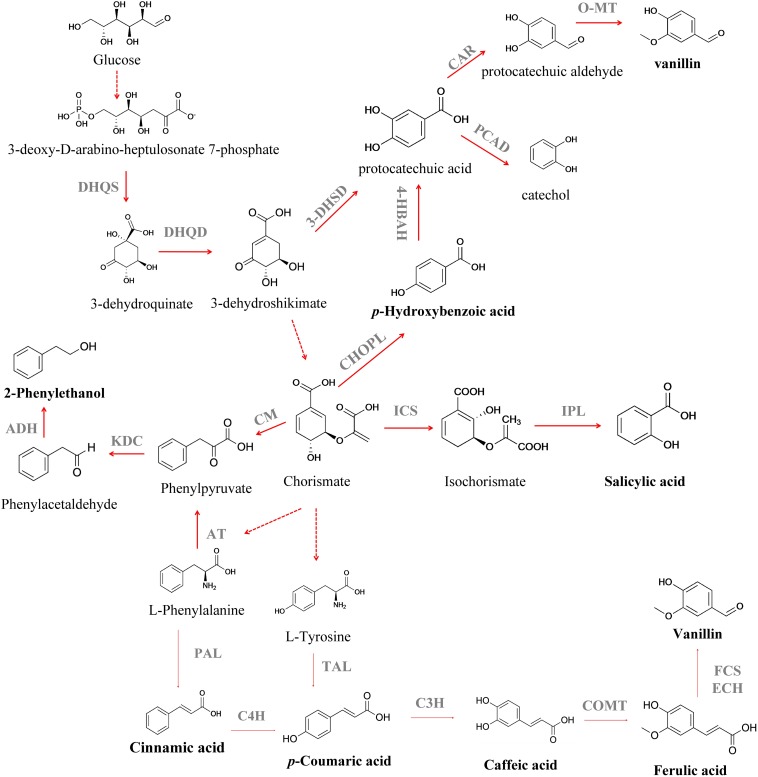
Biosynthesis of different aromatic compounds derived from the extended shikimate pathway. DHQS, 3-dehydroquinate synthase; DHQD, 3-dehydroquinate dehydratase; 3-DHSD, 3-dehydroshikimate dehydratase; CAR, carboxylic acid reductase; PCAD, protocatechuic acid decarboxylase; O-MT, *O*-methyltransferase; ICS, isochorismate synthase; CHOPL, chorismate pyruvate lyase; IPL, isochorismate pyruvate lyase; 4-HBAH, 4-hydroxybenzoic acid hydroxylase; CM, chorismate mutase; KDC, phenylpyruvate decarboxylase; ADH, alcohol dehydrogenase; PAL, phenylalanine ammonia lyase; C4H, cinnamate 4-hydroxylase; TAL, tyrosine ammonia lyase; FCS, feruloyl-CoA synthetase; ECH, feruloyl-CoA hydratase/lyase; C3H, *p*-coumarate 3-hydroxylase; AT, aminotransaminase. Solid lines indicate a single step; dotted lines indicate multiple steps.

Salicylic acid (SLA) (2-hydroxybenzoic acid) is a valuable aromatic compound that can be obtained from CHO (Lin et al., 2014; Jiang and Zhang, 2016). SLA is an important drug precursor mainly used to produce acetylsalicylic acid, widely applied as a non-steroidal anti-inflammatory drug, in the treatment of fever, pain, aches and inflammations (Vane and Botting, 2003). Isochorismate synthase (ICS) converts CHO to isochorismate and then isochorismate pyruvate lyase (IPL) converts isochorismate into SLA (Serino et al., 1997; Figure 2). Lin et al. (2013) attained an SLA titer of 158 mg L^–1^ in *E. coli* after the expression of *entC* from *E. coli* (ICS step) and *pfpchB* from *P. fluorescens* (IPL step), as an operon. Lin et al. (2014) further improved the metabolic flux toward SLA using a medium copy number plasmid, pCA-APTA, to express *aroL*, *ppsA*, *tktA* and *aroG*^fbr^, under the control of an IPTG-inducible promoter (P_L_lacO1), attaining an SLA titer of 1.2 g L^–1^, using glycerol as carbon source. Noda et al. (2016) reported the highest SLA titer to the date, 11.5 g L^–1^ (Table 1), with a yield of 41.1 % from glucose, after enhancing the availability of PEP in *E. coli*. They removed the endogenous PEP consuming PTS, that was replaced by GalP/Glk system, as well as the genes responsible for the conversion of PEP to pyruvate (*pykF* and *pykA*). Finally, the strain was further modified by the introduction of *menF* from *E. coli* (ICS step) and *pchB* from *P. aeruginosa* (IPL step). In that report, an 8-fold increase in SLA concentration (from 1.4 to 11. 5 g L^–1^) was attained after the process scale up to 1-L jar fermenter. These findings demonstrate the importance of balancing the plasmid copy number and the impact of deleting the genes from SKM pathway in cell growth and production titers. However, the SA toxicity toward the producing cell remains a challenge, and it is necessary to develop more resistant strains or explore alternative chassis that naturally exhibit high tolerance toward toxic compounds, as *Pseudomonas aeruginosa* (Jiménez et al., 2002; Nikel and de Lorenzo, 2018). The microbial production of *p*-hydroxybenzoic acid (PHBA) can also be achieved from CHO by chorismate pyruvate lyase (Figure 2). This aromatic compound is used as a building block for liquid crystal polymers and for antibacterial parabens – a key group of compounds used as food preservatives (Barker and Frost, 2001), with an estimated market value of $150 million per year (Krömer et al., 2013). Nowadays, PHBA is chemically synthesized from benzene via cumene and phenol (Heinz-Gerhard and Jurgen, 2012). The biotechnological production of PHBA has already been described in plants, like tobacco (*Nicotiana tabacum L.)* and potato (*Solanum tuberosum* L.) (Köhle et al., 2003), in *E. coli* (Barker and Frost, 2001), *Klebsiella pneumoniae* (Müller et al., 1995), *C. glutamicum* (Kallscheuer and Marienhagen, 2018), and *P. putida* (Verhoef et al., 2007), using glucose as carbon source or with complex mixtures such as sugar cane (Mcqualter et al., 2005). Barker and Frost (2001) reported PHBA production in *E. coli* after overexpression of *aroF*^fbr^ (feedback-inhibition resistant DAHP synthase), as well as the genes involved in the SKM pathway (*tktA*, *aroA*, *aroL*, *aroC*, and *aroB*) and encoding chorismate pyruvate lyase (*ubiC*, that was expressed in a plasmid under the control of a tac promoter). A PHBA yield of 12 g L^–1^ was obtained in a fed-batch fermentation. The application of an *E. coli*–*E. coli* co-culture system was recently reported by Zhang H. et al. (2015) for PHBA production from glucose and xylose, a sugar mixture that can be derived from lignocellulose. The authors reported a 8.6-fold improvement in PHBA production when its biosynthesis was switched from the monoculture strategy to the co-culture strategy. Finally, a fed-batch bioreactor was used to scale up the PHBA production and under this new condition its titer was improved to 2.3 g L^–1^. The production of this aromatic compound in yeast was described for the first time by Krömer et al. (2013). The authors reported a PHBA titer of 90 mg L^–1^ in *S. cerevisiae* after overexpression of an *ubiC* from *E. coli* and deletion of *ARO7* and *TRPp3*, avoiding the biosynthesis of aromatic amino acids. To increase the flux to chorismate, they expressed *ARO4*^K229L^ and *aroL*. This strain was then used and allowed a PHBA formation from CHO, with a titer of 2.9 g L^–1^ and yield of 3.1 mg g_glucose_^–1^, in a fed-batch process (Averesch et al., 2017). To date, the highest PHBA titer was reported by Kitade et al. (2018) in *C. glutamicum* (Table 1). This was achieved by chromosomal integration of *aroG* from *E. coli* and wild-type *aroCKB* from *C. glutamicum*, encoding chorismate synthase, shikimate kinase, and 3-dehydroquinate synthase. In order to convert CHO to HPBA a highly HPBA-resistant chorismate pyruvate lyase (encoded by *ubiC*) from the intestinal bacterium *Providencia rustigianii* was used. In order to increase product formation, the synthesis of by-products was also reduced by deleting *hdpA* and *pyk*. The final strain produced 36.6 g L^–1^ of PHBA from glucose after 24 h, with a glucose yield of 40 % (mol mol^–1^). Despite the efforts that have been made to increase the production yields and concentration of PHBA, the obtained results still fall behind those benchmarks from an industrial perspective. In fact, PHBA itself could also be toxic to the cells at high concentrations and the application of *in situ* product removal (ISPR) strategies will lead to a continuous PHBA removal from the fermentation broth. An interesting approach was presented by Johnson et al. (2000) that applied an *in situ* product removal technique for PHBA production with *E. coli* using Amberlite IRA-400 as adsorbent, and observed an increase in the PHBA titer from 6 to 22.9 g L^–1^. In the future, further improvements in the biotechnological process will be necessary, such as the utilization of low-cost substrates, as residues and wastes, as well as the development of a “green” downstream process, to pique the industry interest in these processes.

Hydroxycinnamic acids are an important class of hydroxylated aromatic acids that contain a phenol ring and at least one organic carboxylic acid group. This group of compounds includes *p*-coumaric acid, caffeic acid and ferulic acid, among others. *p*-Coumaric acid is an important platform chemical used as monomer of liquid crystal polymers for electronics (Kaneko et al., 2006), as well as precursor for the synthesis of polyphenols (Rodriguez et al., 2015). The route for their production starts with L-Phe and L-Tyr deamination to further produce the phenylpropanoid cinnamic acid and *p*-coumaric acid, respectively, by the activity of phenylalanine ammonia lyase (PAL) (MacDonald and D’Cunha, 2007) and tyrosine ammonia lyase (TAL) (Nishiyama et al., 2010; Figure 2). The enzyme P450 monooxygenase cinnamate 4-hydroxylase (C4H) can further oxidize the cinnamic acid yielding the *p*-coumaric acid (Rasmussen et al., 1999; Achnine et al., 2004). The heterologous expression of TAL and PAL/TAL encoding genes allowed the *p*-coumaric acid production in *E. coli*, *S. cerevisiae*, *Streptomyces lividans* and *P. putida* (Table 1; Nijkamp et al., 2007; Trotman et al., 2007; Vannelli et al., 2007; Kawai et al., 2013; Rodriguez et al., 2015; Vargas-Tah and Gosset, 2015). However, due to their low activity, first studies reported its production from culture medium supplemented with L-Phe or L-Tyr (Ro and Douglas, 2004; Hwang et al., 2003; Watts et al., 2004; Jendresen et al., 2015; Mao et al., 2017). Efforts have also been made to find enzymes from different sources with higher PAL or TAL activity (Nijkamp et al., 2005, 2007; Vannelli et al., 2007; Kang et al., 2012; Jendresen et al., 2015). *p*-Coumaric acid production from a simple carbon source, such as glucose, is desirable. In order to achieve this, *S. cerevisiae* was genetically modified (Vannelli et al., 2007). The encoding PAL/TAL gene from *Rhodotorula glutinis* (expressed under the control of the galactose promoter) was used due to its higher affinity toward L-Tyr compared to L-Phe. The heterologous expression of a C4H gene from *Helianthus tuberosus* allowed its production via the PAL route. More recently, Li et al. (2018) proposed the *p*-coumaric acid production from glucose via phenylalanine in *E. coli*. They expressed the C4H-encoding gene from *Lycoris aurea* and PAL1 of *Arabidopsis thaliana*, under a trc promoter induced by IPTG, attaining a titer of 25.6 mg L^–1^ in shake flasks, after the regulation of the intracellular level of NADPH. The authors observed that the level of intracellular NADPH has a strong impact on the conversion of *trans*-cinnamic acid into *p*-coumaric acid and different strategies were tested in order to increase the level of intracellular NADPH. When *pntAB*, that encodes a membrane-bound transhydrogenase that catalyzes the NADH to NADPH conversion, was overexpressed under the control of a T7 constitutive promoter and the synthetic small regulatory RNA (srRNA) anti(SthA) was used to specifically repress the translation of the soluble transhydrogenase SthA, a synergetic positive effect was observed on the *de novo* production of *p*-coumaric acid. To date, the highest *p*-coumaric acid titer, 2.4 g L^–1^, has been achieved in *S. cerevisiae* after overexpression of the encoding TAL gene from *Flavobacterium johnsoniae;* overexpression of *aroL* from *E. coli*, under control of the P-TEF promoter; overexpression of *Aro7*^G141S^ and *Aro4*^K229L^ from *S. cerevisiae* under control of the promoters P-TEF and P-PGK1, respectively and deletion of *Aro10* and *Pdc5* genes (Rodriguez et al., 2017; Table 1). However, it is also important to explore the application of other raw materials derived from biomass as carbon sources – see section “Discussion.” Vargas-Tah and Gosset (2015) managed to produce cinnamic acid and *p*-coumaric acid in *E. coli* using lignocellulosic hydrolysates as complex carbon source. However, other strains that grow naturally on complex carbon sources were also used in the production of these hydroxycinnamic acids, such as *Streptomyces lividans* (Noda et al., 2011, 2012; Kawai et al., 2013) with product concentrations ranging from 130 to 736 mg L^–1^. In the last year, Borja et al. (2019) constructed a *S. cerevisiae* strain that uses xylose as sole carbon source for *p*-coumaric acid production, attaining a titer of 242 mg L^–1^, that represents a 45-fold increase over their condition with glucose (5.25 mg L^–1^) (Table 1). To construct this strain, they knocked out *Aro10* and *Pdc5* genes, in order to reduce the byproduct formation, and overexpressed the encoding TAL gene from *F. johnsoniae* and *aroL* from *E. coli.* To increase the carbon flux through the aromatic amino acid pathway they overexpress *Aro7*^*fbr*^ (feedback-inhibition resistant DAHP synthase) and *Aro4*^fbr^ (feedback-inhibition resistant chorismite mutase). Another important issue detected in these studies is the toxic effect of *p*-coumaric acid to the producing cells. An alternative strategy was proposed by Huang et al. (2013), that involves pulse feeding to avoid the accumulation of *p*-coumaric acid to a toxic level. The resistance to toxic compounds can also be increased using membrane transport engineering as a method to decrease the intracellular concentration of a toxic compound. In *E. coli*, the overexpression of *aaeXAB* gene, that encodes an efflux pump for several aromatic compounds, resulted in a twofold increase in tolerance to *p*-coumaric acid (Dyk et al., 2004; Sariaslani, 2007). Caffeic acid is another important intermediate of the phenylpropanoid metabolism. It serves as a precursor for the synthesis of caffeoyl alcohol and 3,4-dihydroxystyrene (monomer for plastic synthesis) (Zhang and Stephanopoulos, 2013). Caffeic acid is biosynthesized by hydroxylation of *p*-coumaric acid through *p*-coumarate 3-hydroxylase (C3H) (Berner et al., 2006; Figure 2). First studies have reported microbial caffeic acid production with medium supplementation of the precursors such as L-Tyr and *p*-coumaric acid (Sachan et al., 2006; Choi et al., 2011). In order to produce it directly from *p*-coumaric acid, it is necessary to express the genes that encode enzymes with suitable ring 3-hydroxylation activity (Berner et al., 2006; Furuya et al., 2012). In fact, one of the major difficulties in the heterologous expression of genes of the plant phenylpropanoid pathway in *E. coli* is the lack of cytochrome P450 reductase activity making the search for alternative strategies essential. Choi et al. (2011) identified that the *sam5* gene from *Saccharothrix espanaensis* encodes C3H, that is a FAD-dependent enzyme. This enzyme was then used to produce caffeic acid in *E. coli*, demonstrating the feasibility of this two-step pathway to produce caffeic acid using alternative enzymes. It was also demonstrated that a bacterial cytochrome P450 CYP199A2, from *Rhodopseudomonas palustris*, was able to efficiently convert *p*-coumaric acid to caffeic acid (Hernández-Chávez et al., 2019). Some microorganisms, harboring genes encoding the two sub-units of the enzyme 4-hydroxyphenylacetate 3-hydroxylase (4HPA3H), have proved to be able to act on aromatic compounds (Galán et al., 2000). It was also observed that 4HPA3H is able to convert *p*-coumaric acid into caffeic acid. Based on this Furuya and Kino (2014) expressed the 4HPA3H gene from *Pseudomonas aeruginosa* in *E. coli* and a caffeic acid production of 10.2 g L^–1^ was obtained after repeated additions of *p*-coumaric acid (20 mM each pulse), in a medium with glycerol as carbon source. For caffeic acid biosynthesis from L-Tyr an additional step of non-oxidative deamination, catalyzed by TAL (Rodrigues et al., 2015) is needed, and the first approach is the search for alternative enzymes from different sources. TAL from *R. glutinis* proved to be the most active TAL identified (Vannelli et al., 2007; Santos et al., 2011). However, the production of this compound from simple carbon sources is much more desirable and the production of caffeic acid from glucose and xylose was described (Lin and Yan, 2012; Zhang and Stephanopoulos, 2013). The use of renewable feedstocks, such as lignocellulosic biomass, was also evaluated using *E. coli* as producing host (Kawaguchi et al., 2017). A maximum caffeic acid concentration of 233 mg L^–1^ (Table 1) was produced from kraft pulp using a tyrosine-overproducing *E. coli* strain harboring the *hpaBC* gene from *P. aeruginosa* and *fevV* gene from *Streptomyces* sp. WK-5344. Ferulic acid is a component of lignocellulose (De Oliveira et al., 2015). This *O*-methylated hydroxycinnamic acid is biosynthesized from caffeic acid by caffeic acid *O*-methyltransferase (COMT) (Figure 2). However, there are few reports about the heterologous production of ferulic acid in microbial hosts. Choi et al. (2011) reported the production of ferulic acid in *E. coli* by expression of *sam5*, a TAL gene from *S. espanaensis* and a COMT gene from *A. thaliana*, under the control of a T7 promoter, attaining a titer of 0.1 mg L^–1^ from L-Tyr. Later, Kang et al. (2012) engineered an *E. coli* strain capable of producing 196 mg L^–1^ of ferulic acid from glucose, after introduction of COMT from *A. thaliana* in a caffeic acid-over-producing strain, containing a codon optimized *tal* gene, under the control of a T7 promoter. This aromatic acid can also be used as substrate for vanillin and coniferyl alcohol biosynthesis (Hua et al., 2007; Lee et al., 2009; Chen et al., 2017). Research efforts have been made in order to make the microbial production of hydroxycinnamic acids a competitive process.

Another important class of aromatic compounds is the aromatic flavors class. The two most popular benzenoid flavors are 2-phenylethanol (2-PE) and vanillin. 2-PE is an aromatic alcohol with a delicate fragrance of rose petals widely used in flavor and fragrances industries (Burdock, 2010; Carlquist et al., 2015). It was recently identified as a potent next generation biofuel (Keasling and Chou, 2008). Furthermore, 2-PE can also be used as raw material to produce other flavor compounds (2-phenylethyl acetate and phenylacetaldehyde) and styrene (Etschmann et al., 2002). Microorganisms can naturally produce 2-PE as part of their amino acid metabolism (Etschmann et al., 2002). This benzoid flavor can be produced through the SKM pathway or via Ehrlich pathway from L-Phe (Albertazzi et al., 1994; Carlquist et al., 2015; Figure 2). Through the Ehrlich pathway, L-Phe is firstly converted to phenylpyruvate by transamination, which is then transformed to phenylacetaldehyde by decarboxylation. Then, the derivative aldehyde is reduced to 2-PE by an alcohol dehydrogenase (Etschmann et al., 2002; Figure 2). This is the fastest pathway to produce 2-PE, however cheaper precursors than L-Phe should be used to achieve a more competitive process (ÄYräpää, 1965; Kim T.Y. et al., 2014; Zhang et al., 2014). Thus, *de novo* production of 2-PE has been described in different microorganisms: *Kluyveromyces marxianus* (Kim T.Y. et al., 2014), *E. coli* (Guo et al., 2018; Liu et al., 2018), and *Enterobacter* sp. (Zhang et al., 2014; Table 1). The 2-PE production from the SKM pathway is achieved from its end product phenylpyruvate, that is decarboxylated to phenylacetaldehyde, followed by a dehydrogenation that leads to 2-PE production (Etschmann et al., 2002; Carlquist et al., 2015; Figure 2).

Nonetheless, the *de novo* synthesis is inefficient since glycolysis and PPP are mainly used for cell growth, producing very low 2-PE concentrations. The most common strategies employed for strain constructions focus on increasing phenylpyruvate decarboxylase and alcohol dehydrogenase activities, which are the rate-limiting enzymes in *de novo* synthesis pathway, in combination with feedback-resistant DAHP synthase and chorismate mutase. Kim B. et al. (2014) overexpressed the *Aro*10, that encodes a transaminated amino acid decarboxylase and *Adh*2, encoding an alcohol dehydrogenase, from *S. cerevisiae* in *K. marxianus* BY25569, under the control of the constitutive promoter *Sc*PGK1/*Sc*TEF1. Then, serial subcultures with an L-Phe analog, *p*-fluorophenylalanine, were conducted in order to obtain an evolved strain resistant to the L-Phe analog. Finally, the expression of *aroG*^fbr^ from *Klebsiella pneumoniae*, that encodes a feedback-resistant mutant of DAHP synthase, was also performed. This genetically modified strain was able to produce 1.3 g L^–1^ of 2-PE from glucose without addition of L-Phe. More recently, Liu et al. (2018) constructed a heterologous pathway from *Proteus mirabilis* in *E. coli* and the recombinant strain was able to produce 1.2 g L^–1^ of 2-PE without L-Phe supplementation (Table 1).

Another challenge in the biosynthesis of 2-PE is its toxicity. Different strategies were investigated in order to improve the process yield and productivity, being the most common the optimization of medium composition, operational conditions and application of ISPR techniques (Chung et al., 2000; Hua et al., 2010; Cui et al., 2011; Celińska et al., 2013; Mihal’ et al., 2014). 2-PE production is highly dependent on media composition and culture conditions (Garavaglia et al., 2007). The utilization of an interesting alternative carbon source was reported by Celińska et al. (2013). They use glycerol as carbon source in bioconversion of L-Phe to 2-PE by *Yarrowia lipolytica* NCYC3825, reaching a 2-PE production of 0.77 g L^–1^ after 54 h. Another recent approach for this flavor production was reported by Martínez-Avila et al. (2018). In the proposed system, *K. marxianus* ATCC10022 used the available nutrients from a residue-substrate (sugarcane bagasse) supplemented with L-Phe, achieving a 2-PE production of 10.21 mg g^–1^ (mass of product per mass of solid) in a fed-batch system. The application of alternative modes of operation, such as fed-batch and continuous, that allow the possible removal or dilution of 2-PE in the medium, are also interesting approaches recently reported. Last year, *de novo* production of 2-PE by *Metschnikowia pulcherrima* NCYC373 reached higher titers in continuous mode operation, than in batch and fed-batch cultures (Chantasuban et al., 2018). In continuous fermentation, 2-PE concentration levels reached 1.5 g L^–1^, before it became too toxic and caused the flush out (Table 1). Even with the efforts to optimize the culture medium and cultivation conditions, and choose the most producing microorganism, product inhibition is still the major problem of 2-PE biosynthesis (Carlquist et al., 2015). Some strategies, such as ISPR techniques, have been developed to reduce the 2-PE toxicity in the fermentation medium, increasing its production (Carlquist et al., 2015). Recently, Chantasuban et al. (2018) reported the application of oleyl alcohol as an extraction phase in the 2-PE production by *M. pulcherrima* NCYC373. The production levels were enhanced with the application of this ISPR technique, achieving a 2-PE concentration of 1.96 g L^–1^ in the aqueous phase and an overall production of 3.13 g L^–1^. Gao and Daugulis (2009) reported a highly significant enhancement in the 2-PE production, using a solid-liquid two-phase partition bioreactor with polymer beads as the sequestering immiscible phase. The batch mode system reached a final 2-PE concentration of 13.7 g L^–1^ (88.74 g L^–1^ in the polymer phase and 1.2 g L^–1^ in the aqueous phase), whereas the fed-batch achieved an overall titer of 20.4 g L^–1^ (97.0 g L^–1^ in the polymer phase and 1.4 g L^–1^ in the aqueous phase). During the last years, great improvements have been achieved in the bioproduction of 2-PE leaving its industrial application closer. In fact, 2-PE concentrations of 21 g L^–1^ were reached (Mihal’ et al., 2014), in an hybrid system that consists of a fed-batch stirred tank bioreactor and a hollow fiber membrane module immersed at the bottom of the bioreactor, where 2-PE is continuously extracted from the fermentation broth using pentane as the organic phase.

Vanillin (4-hydroxy-3-methoxybenzaldehyde), a widely used flavor compound in different industries, is the primary component of the extract of the vanilla bean. The economic importance of this plant natural product is quite significant; it was reported that synthetic vanillin has a price of around US$ 11 kg^–1^, while biotech vanillin is sold for a price of around US$ 1000 kg^–1^ (Schrader et al., 2004). Over the last years, vanillin production through biotransformation of ferulic acid, isoeugenol, lignin, was reported, with vanillin titers that range from 0.13 to 32.5 g L^–1^ (Huang et al., 1993; Priefert et al., 2001; Zhao et al., 2005; Kaur and Chakraborty, 2013). Furuya et al. (2015) reported a vanillin concentration of 7.8 g L^–1^ from ferulic acid in a two-stage process with an *E. coli* carrying two expression plasmids harboring *fdc* from *Bacillus pumilus* and *cso2* from *Caulobacter segnis*. However, the vanillin production through these pathways has several bottlenecks that include the price of precursors, the formation of undesired side-products and the cytotoxicity of the precursors (Gallage and Møller, 2015). Based on this, its production by *de novo* biosynthesis from cheap and more available carbon sources is much more attractive.

In the pathway from 3-dehydroshikimate (3-DHS) to vanillin, the first step is the dehydration of 3-DHS to protocatechuic acid, catalyzed by 3-dehydroshikimate dehydratase, that is further converted to protocatechuic aldehyde, by carboxylic acid reductase; and, the final step is catalyzed by an *O*-methyltransferase leading to vanillin (Hansen et al., 2009; Figure 2). Li and Frost (1998) were the first to report an engineered pathway for vanillin production in *E. coli.* Here, protocatechuic acid was converted to vanillic acid by catechol-*O*-methyltransferase, and further reduced to vanillin by an aryl aldehyde dehydrogenase. Hansen et al. (2009) explored for the first time the vanillin production in the yeasts *Schizosaccharomyces pombe* and *S. cerevisiae.* The authors introduced a DHS dehydratase (3DSD) from *Podospora pausiceta*, an aromatic carboxylic acid reductase (ACAR) from *Nocardia* sp., a phosphopantetheinyl transferase (PPTase) from *C. glutamicum* to activate ACAR, and an *O*-methyl transferase (OMT) from *Homo sapiens*, allowing a vanillin production of 45 mg L^–1^. More recently, Kunjapur et al. (2014) used an *E. coli* strain with decreased aromatic aldehyde reduction activity as a host for the biosynthesis of vanillin. A vanillin titer of 119 mg L^–1^ was achieved using an *E. coli* strain expressing a *Bacillus thuringiensis* 3-dehydroshikimate dehydrogenase gene (*asbF*), a *H. sapiens O*-methyltransferase gene (*Hs-S-COMT)* and *Nocardia iowensis* carboxylic acid reductase gene (*car*), that are codon optimized and expressed in a plasmid. They also introduced a feedback-resistant DAHP synthase (encoded by *aroG*) and a phosphopantetheinyl transferase (encoded by *sfp*) from *B. subtilis*, which have been shown to activate CAR.

However, *de novo* vanillin production in recombinant bacteria and yeasts still has challenges that are not only related with product formation itself but also the product toxicity. The major hurdle in the biotechnological production of vanillin is the strong inhibitory effect that this flavor has on microorganism growth (Gallage et al., 2014; Ma and Daugulis, 2014). An interesting approach was proposed by Brochado et al. (2010), in which the natural pathway for vanillin production in plants was mimicked and assembled in *S. cerevisiae*. To overcome the toxicity of vanillin, a gene encoding an uridine diphosphate–glucose glycosyltransferase (UGT) from *Arabidopsis thaliana* was expressed in *S. cerevisiae.* This UGT catalyzes the glycosylation of vanillin and produces a less toxic final product, vanillin-β-d-glucoside (VG). The same strategy was implemented by Ni et al. (2015) allowing a VG production of 500 mg L^–1^, with a yield of 32 mg g_glucose_^–1^, that is 5-fold higher than the 45 mg L^–1^ reported by Hansen et al. (2009). A different strategy was presented by Yoon et al. (2007) for vanillin production from ferulic acid using an *E. coli* strain harboring a plasmid with *fcs* (feruloyl-CoA synthase) and *ech* (enoyl-CoA hydratase/aldolase) genes from *Amycolatopsis* sp. strain. To reduce the vanillin toxicity, they improved the vanillin-resistance of this strain using NTG mutagenesis as well as a XAD-2 resin to remove the vanillin from the medium. When 50 % (w/v) of XAD-2 resin was used with 10 g L^–1^ of ferulic acid, the vanillin production with the NTG-VR1 mutant strain was 2.9 g L^–1^, which was 2-fold higher than that obtained without resin. Recently, Luziatelli et al. (2019) reported, for the first time, the vanillin production from ferulic acid using a plasmid free *E. coli* strain, after chromosomal integration of *fcs* and e*ch* genes from *Pseudomonas*. In addition, they also performed an optimization of the bioconversion conditions (namely stirring speed and initial substrate concentration) using a response surface methodology. At the same time, the authors used a two-phase (solid-liquid) system where the substrate was incorporated in a gel matrix (agarose-gel) in order to perform a fixed volume fed-batch approach, for controlled release of ferulic acid. Using this two-phase system, a vanillin titer of 4.3 g L^–1^ was attained in the liquid phase – one of the highest found in the literature for recombinant *E. coli* strains (Table 1).

## Discussion

The production of aromatic compounds through plant extraction or chemical synthesis is a profitable business that’s been in place for quite a few years, now. In order to replace these industrial processes by fermentation based biotechnological processes, these must have clear economic, environmental and/or product wise advantages. From an economic point of view, the advantages have to be significant enough to justify the investment on new industrial equipment.

In general, these processes have a lower environment impact and are able to produce high quality final products when compared to their plant extraction and/or chemical synthesis counterparts (Thompson et al., 2015; Dudnik et al., 2017; Kallscheuer et al., 2019).

One of the advantages of fermentation based processes is the low-cost and abundance of the raw materials – low added value sugars. However, in order to achieve this advantage, the processes must not consider the supply of expensive precursors, antibiotics, inducers, etc… The optimization of the microorganisms in order to produce the desired aromatic compound directly form the substrate is usually a requirement to achieve economic feasibility. This is supported by Li et al. (2018) and Liu et al. (2018) studies – among other examples previously presented (Table 1) – showing that the strain engineering toward deregulation of the aromatic amino acids metabolism and an optimal connection of the heterologous pathways to the host metabolism enable aromatic compounds production starting from glucose without any need for supplementation of precursor metabolites.

In recent years, some research effort has been put into replacing the hydrocarbon source for these fermentation processes by residues and waste materials (Vargas-Tah and Gosset, 2015; Martínez-Avila et al., 2018; Borja et al., 2019). Sugars like glucose are abundant low-cost raw materials. On the other the use of waste materials in large scale industrial processes implies assuring a reliable constant supply and usually a pre-treatment step that may add a significant cost to the process. Nevertheless, whenever there is a need to process these wastes in order avoid their environmental impact, a fermentation process that converts them to higher added value products is an alternative worth considering.

Another possible bottleneck for the industrialization of these processes is the cost of the purification step. Microorganisms tend to produce a mixture of by-products where the compound of interest may be in higher or lower concentration. Although the amount of research put into this field is not always as significant as one would expect, the search for higher titers and the use of ISPR techniques has direct impact on the economic sustainability of these processes (Van Hecke et al., 2014). Moreover, it will also be interesting to study the synergetic application of engineered strains, able to use wastes and residues as substrates, in systems using ISPR techniques, as well as the product recovery and purification (Braga et al., 2018b; Kallscheuer et al., 2019).

Different microorganisms have been used to produce aromatic compounds. The most commonly used hosts are *E. coli*, *S. cerevisiae* and *C. glutamicum*. However, tolerant and thermotolerant microorganisms, as well as a broad spectrum of bacteria were also employed as aromatic compound producers (Table 1). The choice of the microorganism employed is always a determinant factor for the outcome of any research project in this field. The exploitation of well established platform organisms, for which metabolic engineering tools are available, is the most common approach. However, it is also important to explore non-model organisms that can naturally produce the desired compounds, even though the available genetic tools are still scarce. For example, the metabolic versatility of *Pseudomonas* as well as it inherent tolerance to toxic compounds, offers an excellent starting point for suppressing the hurdles of using and producing toxic compounds of natural or heterogeneous origin (Krömer, 2016; Lenzen et al., 2019; Table 1).

Some aromatic compounds are toxic for the producing host. In order to tackle this obstacle, several alternative strategies have been proposed such as: starting the fermentation with a lower substrate concentration and further additions following its consumption rate (step-wise fed-batch); the use of *in situ* product removal strategies and the application of adaptive evolution to obtain strains with enhanced resistance to toxic products. Aiming at industrial scale operation, the reduction of the production costs is always crucial. In order to reduce the medium cost, chromosomal integration of heterologous genes avoid the use of expensive antibiotics and inducers for plasmid maintenance and inducible expression (Cui et al., 2014).

Nowadays, the microbial production of aromatic compounds is already implemented at industrial scale in economically viable process. Evolva, for instance, has launched a process for vanillin production from glucose with a genetically modified *Schizosaccharomyces pombe* (Vanilla, 2014). Similarly, Solvay has a process for vanillin production with *Streptomyces setonii* from ferulic acid (Muheim et al., 2001). In these cases, the product titers attained are in the order of a hundred g *per* L, and the methodologies for product separation and purification are well established. However, despite the efforts that have been made to increase the production titers with microbial hosts, the concentrations obtained for more complex compounds are still too low (mg and μg *per* L) for an industrial process (Table 1), and these molecules are still produced by extraction from natural sources or by chemical synthesis. In fact, it has been predicted that the market value of a bulk chemical is less than $10 kg^–1^. On the other hand, fine chemicals are produced in limited volumes (<1000 tons per year) but at relatively high prices (>$10kg^–1^) (Joshi and Ranade, 2016). Based on this, at least for now, the commercialization of fine aromatic compounds will emerge with more successes (Cao et al., 2019). The current challenges that still need to be addressed are the metabolic imbalances in the producer strain, the availability of the metabolites required for biomass formation and the product extraction and purification. In a near future it can be expected that with the application of novel synthetic biology approaches, such as CRISPR/Cas9, rational strain engineering, adaptive laboratory evolution and high-throughput screening approaches, it will be possible to render the microbial production of additional aromatic compounds and its derivatives economically viable.

## Final Remarks

Production cost is, by far, the main obstacle to overcome in order to industrialize the production of aromatic compounds through fermentation. This explains the two main research goals mentioned throughout this review: the maximization of product titers and the removal of expensive fermentation media ingredients.

The main challenges to address are still the low availability of precursor molecules by the microbial metabolism, the elimination of complex pathway regulations, disruption of competing pathways and the low activity of heterologous enzymes in the microbial hosts. It is expected that with recent technological innovations the engineering of microbial host strains will be faster providing more precursor molecules to increase aromatic compound synthesis. Identification of the most suitable enzymes and their further improvement will also be an important step toward the production of different aromatic chemicals, avoiding the accumulation of undesired intermediates that can be toxic to the microbial host and leading to an increase in the final product titer. Metabolic engineering, system and synthetic biology tools for strain design, together with process engineering strategies have been and will continue to be the main resources applied. In addition, the identification of novel enzymes that catalyze non-natural reactions, or novel synthetic pathways not found in nature, allow the production of the desired molecule with a high yield or a non-natural compound with possibly superior or new therapeutic properties.

Overall, the paradigm moves toward the development of better microbial chassis and new metabolic pathways that allow the shift from producing aromatic compounds from fossil resources to a bio-based production. The authors consider that now, the biotechnological production of aromatics is not a question of whether or not it is theoretically possible, but of when will it become technically and economically feasible.

## Author Contributions

AB and NF contributed to the conception and design of the study. AB reviewed the literature, extracted the data, and drafted the manuscript. NF edited the manuscript. Both authors read and approved the submitted version.

## Conflict of Interest

The authors declare that the research was conducted in the absence of any commercial or financial relationships that could be construed as a potential conflict of interest.
